# Examining Intersectional Role of Race, Ethnicity, and Sexual Orientation in Health Disparities Among Older Adults

**DOI:** 10.1093/geroni/igaf122.3421

**Published:** 2025-12-31

**Authors:** Hyun-Jun Kim, Hailey Jung, Austin Oswald, Karen Fredriksen-Goldsen

**Affiliations:** University of Washington, Seattle, Washington, United States; University of Washington, Seattle, Washington, United States; Dalhousie University, Halifax, Nova Scotia, Canada; University of Washington, Seattle, Washington, United States

## Abstract

Lesbian, gay, and bisexual (LGB) older adults who are racially/ethnically minoritized may face heightened health disadvantages due to their combined impact inclusive of the risks due to each social position and any excess risks beyond (i.e., synergistic effect), yet empirical evidence remains scarce. To address this gap, we used the 2012-2023 National Health Interview Survey to examine intersectional health disparities among Hispanic, Black, Asian, and other individuals of color within LGB adults age 50+ in the United States. Using non-Hispanic White heterosexuals as the referent and stratifying by gender, we estimated joint health disparities by sexual orientation and race/ethnicity and tested synergistic effects while adjusting for age, education, and income. Among older women, we observed joint disparities, predominately driven by synergistic effects, in cardiovascular disease for other LGB individuals of color (risk difference [RD] 15.5 percentage points [pp], 95% CI 5.5-25.6). Among older men, joint disparities, largely driven by synergistic effects, were observed in asthma (6.1 pp, 95% CI 0.0-12.2) and mental distress (16.4 pp, 5.5-27.3) for Hispanic LGB individuals; cognitive (12.7 pp, 3.0-22.4) and vision impairments (3.4 pp, 3.7-23.1) for Black LGB individuals; and limited physical functioning (15.1 pp, 3.2-26.9), anxiety (28.8 pp, 12.8-44.9), depression (24.5 pp, 8.4-40.5), and poor general health (21.9 pp, 8.1-35.7) for other LGB individuals of color. Overall, health disparity patterns in LGB older adults varied across race/ethnicity, lending some support for the synergistic multiple disadvantage hypothesis. There is a need for future research on intersectionality to identify modifiable mechanisms to eliminate health disparities.

